# An Illustration of Interpersonal Psychotherapy for Perinatal Depression

**DOI:** 10.1155/2020/8820849

**Published:** 2020-10-07

**Authors:** Huey Jing Renee Tan

**Affiliations:** Department of Psychiatry and Mental Health, Kajang Hospital, Jalan Semenyih, 43000 Selangor, Malaysia

## Abstract

This is a case of a 38-year-old married woman presenting with major depressive disorder one month after the birth of her third child. The depressive episode began in the context of interpersonal difficulties with her husband. In addition, she was also battling an internal conflict of continuing to pursue her career dream as an obstetrician and fulfilling her responsibility as a wife and a mother. Interpersonal psychotherapy (IPT) was selected as the treatment choice as an evidence-based peripartum treatment that could specifically address the two presenting problem areas, i.e., marital interpersonal dispute and role transition. This paper provides an illustration of IPT sessions conducted with verbatim selections of the sessions.

## 1. Introduction

Interpersonal psychotherapy (IPT) is an evidence-based treatment with specific applications for perinatal mood disorders that focuses on interpersonal conflict, role transition, and grief or loss. IPT addresses these problem areas by widening the social network for support through improving communication and role play of practical adaptive response [1].

IPT was developed in the 1970s as a manualized psychotherapy treatment for research purposes and thereby has the advantage of decades of research data to contribute to our theoretical understanding of the onset of psychopathology and mechanism of change [2]. IPT is based on three theoretical frameworks, i.e., attachment theory, interpersonal theory, and social theory. IPT incorporates common principles of psychotherapy such as empathy and problem-solving skills. [2–5].

IPT is an effective, transdiagnostic treatment for psychiatric disorders with specific reference to treatment of major depressive disorder [6–11]. IPT understands that major depression is a medical disorder and that significant life events and poor social support contribute to the onset of the disorder [4, 12]. This case illustrates IPT treatment for major depressive disorder with perinatal onset.

## 2. Case Presentation

This is a case of a 38-year-old lady with three children. She presented with perinatal onset of major depressive disorder reporting symptoms of depressed mood, irritability, helplessness, anhedonia, and insomnia. These symptoms started about 4 weeks following her delivery and had persisted for 6 weeks prior to consultation. There were no previous depressive episodes. She scored 19 on Patient Health Questionnaire-9 (PHQ-9) indicating moderately severe depression. She described depressive ruminations and fatigue from juggling multiple roles as wife, mother, daughter, and daughter-in-law. However, she was afraid to talk to her family about her stressors for fear of overinvolvement of her in-laws. Consistent with perinatal depressive symptomatology, she endorsed ego-dystonic thoughts about dropping the baby, but she was assiduously careful when tending her baby. There was no suicidal ideation.

Prior to her marriage, she was working as a medical officer in the medical department in a hospital. She gave up clinical work 5 years ago to do administrative work in the hospital, because the nature of her work requires night shift availability and her husband and in-laws did not approve of her working night shift. However, she was bored with mundane administrative work. While her husband was advancing in his career, she felt left behind in her desire for professional development. She felt guilty for wanting to pursue her career and to think that her children were the reason for her to feel dissatisfied with life compounding her guilty ruminations. She had been thinking about returning to clinical work for some time, however, was fearful to make the change. Meanwhile, she had difficulty communicating these emotions to her husband due to her fear that he would not be able to understand her and give her emotional support.

### 2.1. Therapeutic Intervention

She presented with symptoms of postnatal depression that was clinically significant. Her symptoms were not part of normal postpartum experiences, because the symptoms worsen over weeks and affected her social function and ability to look after her children. In addition, there were associated infanticide ideations. There was no comorbidity. Pharmacological treatment was thus warranted and Escitalopram was initiated and optimized to 20 mg ON.

Psychological intervention specifically interpersonal psychotherapy was also offered to the patient. She was a suitable candidate for interpersonal psychotherapy, because she was aware of her internal emotions and thoughts and was motivated to make changes to improve her situation. A collaborative IPT approach was initiated to formulate her presenting problems with a focus on interpersonal dispute and role transition.

### 2.2. Interpersonal Psychotherapy

#### 2.2.1. Session 1

The first session of IPT involved assessment of attachment and communication style as well as reviewing the social support network via the use of interpersonal inventory (IPT-I). The patient was invited to write down names of people in their social network in the IPT-I. Those whom the patient felt closer to or had provided more support were in the inner circle. Then, the patient was invited to talk about each of the person in the circle, especially with regard to interpersonal incidents, likes, and dislikes as well as challenges in the relationship. These interpersonal details provide important information about her needs, pattern of coping, and attachment style.

From the IPT-I, the patient's husband and mother were identified as closest to the patient, because there were in the inner circle of IPT-I. The patient described her mother as a very understanding person whom she was able to confide in. She found out that her mother also suffered from postpartum depression and her mother had shared her experience of coping with the disorder. Her children, cousin, and best friend were in the middle circle of the IPT-I. She felt comfortable to confide in her cousin and best friend about her difficulties, because they were both working mothers, thus allowing them to understand her situation easily. Her father was in the outer circle. She was fond of her father who was outspoken and friendly. However, she did not have a habit of confiding in his father for her emotional difficulties. Her in-laws were outside the circle. They often interfered with decisions about childcare, and this made her feel stressed and frustrated. Her newborn was also outside the circle. She felt distant from her, and this had inflicted guilt in the patient.

**Figure 1:**
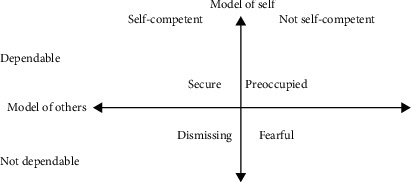
Attachment style in IPT.

An initial assessment concluded that the patient had a fundamentally secure attachment style but became dismissive when under stress. Prior to this peripartum depressive episode, the patient was able to communicate her needs effectively and feel supported by her husband. However, due to recurrent disappointments when her husband was not able to understand her needs, she finds herself reluctant to talk to her husband lately. When under stress, she preferred to isolate herself, particularly from her husband because she perceived him as undependable for her needs. She perceived herself as competent to deal with her stress alone even during the peripartum period. This is an indication of a dismissive attachment style (Figure 1) [2].

#### 2.2.2. Session 2

**Figure 2:**
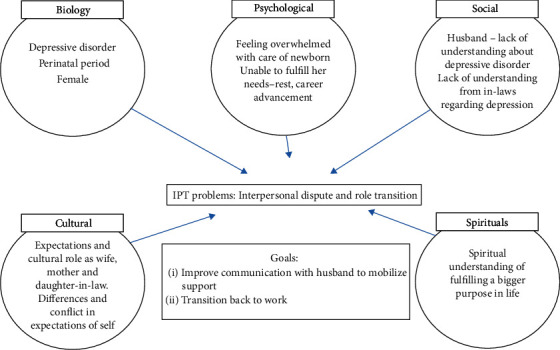
Interpersonal psychotherapy formulation.

In the following session, an IPT summary was formed collaboratively with the patient to help her understand the various factors that contributed to her psychological distress and develop specific goals for the brief treatment (Figure 2). IPT problem areas were identified as interpersonal dispute with her husband and also role transition. She was battling with an internal conflict of her role as a mother, wife, and modern career woman who was keen to excel in her career causing her guilt and dissatisfaction with life. She also faced interference from her in-laws who held a traditional view regarding the role of women in marriage. Although she liked clinical work, she was worried that she might not be able to regain her skills, because she had lost touch with clinical work for more than 5 years. In addition, she was also worried about not being able to fulfill her responsibilities as a wife and mother if she returned to clinical work.

#### 2.2.3. Session 3

**Figure 3:**
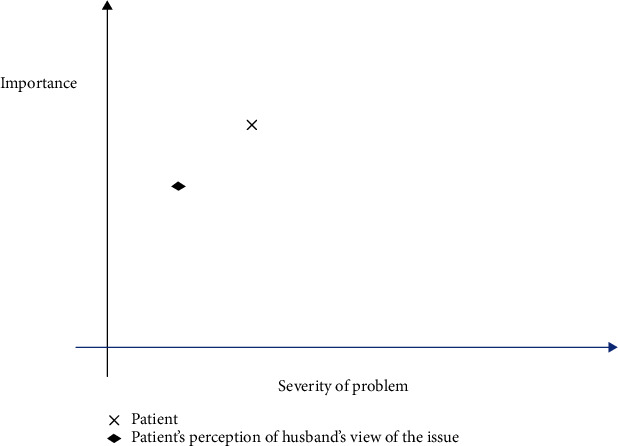
Interpersonal dispute graph.

The interpersonal dispute graph was used to help her with her communication with her husband and to gain his support. It provided a visual gauge on the difference between her perception of the problem and her perception of her husband's view regarding the problem and helped her to reassess the perceived severity of the problem. From the interpersonal dispute graph, she realized that the problem was not as overtly severe. The point of intersection of severity and importance of the problem noted on the graph did not even cross midway on the severity axis (Figure 3). She was concerned about her husband's perception of depression as a sign of weakness and overthinking. We agreed to invite her husband to a session for psychoeducation and discussion about her difficulties. Her husband's willingness to accompany her for the session gave her hope that the problem could be dealt with. Her husband gained more understanding about her illness after he was educated in the session about her symptoms, the contributing factors, and the treatment of depression. This was helpful in bridging the gap between their differences in perception of the problem.

#### 2.2.4. Session 4

Further exploration of interpersonal incidents revealed that the patient was having difficulty having her husband help with the care of their baby. She noticed her husband did not seem to know what to do when the baby cries and would quickly pass the baby to her. Role play on future similar scenario was done during consultation session. Verbatim of the role play is as follows:

Patient (as husband): Dear, baby is crying again. I think you need to take a look.

Therapist (as patient): My dear (baby). There, there. Looks like the diaper is wet. Do not worry, daddy's is going to make you feel better very soon. First, daddy will get you off this diaper …..

#### 2.2.5. Session 5

She reported in the session that she tried out the role play of the previous session and was pleased that her husband responded well. Her depressive symptoms were greatly improved. She scored 1 on PHQ-9. She was beginning to consider returning to clinical work, but she was not feeling confident in her skills due to her 5-year gap of employment. She attempted to tell her husband about her worries, but his response did not validate her fear. She felt pressured by him to make a decision on her career choice and return to work soon. Collaboratively, an analysis of this key interpersonal incident was accomplished and a more adaptive style of communication was role played during the therapy session. In therapy session, the therapist found out that the patient's husband had some worries about returning to fieldwork after being assigned a desk job for a long time. During role play, the therapist (as patient) tried to help her husband (patient) understand her fear by using his own experience of returning to fieldwork as an example.

#### 2.2.6. Session 6

She reported that she was able to have the conversation with her husband just as what was role played in the previous session. She was surprised and pleased to see that her husband was now able to understand her fear. In addition, her husband reassured her about her ability to return to clinical work. However, she continued to have mixed feelings about a career role transition.

An important IPT tool for role transition work is the IPT timeline. This was used in this session to better understand the history of her aspirations during the early years of her career and the role transition she made after she got married. This helped the patient to realize that she could think about her career more flexibly and perhaps offer her service in a general practice or a free clinic and did not have to give up her career as a doctor entirely. Nevertheless, the patient still doubted her clinical skills. The role transition timeline was used to revisit her first day of work as a doctor where she had to work in a new environment far away from home. It helped her to explore how she had coped with the situation. It was suggested that the same coping method can be used should she return to clinical work. Verbatim of the session is provided below.

Verbatim of session 6

Key:

T: Therapist

P: Patient

T: Hi, How are you?

P: I'm better. I think I'm better. So I told my husband when I came back from the session the other day that I have to talk to him.

T: That sounds like a good start.

P: Yeah. It feels quite good after talking to him. He said he did not understand what it meant to me to go back to clinical work but he felt that I can do it. Knowing that he has confidence in me, it gives me confidence and comfort.

T: That's great. So, having that conversation with him was helpful. So if we go back to your goals of returning to clinical work and at the same time still have time for your children. Do you have any thoughts on how you would be able to achieve that?

P: I started out with such high aspirations. But now, a change in career and there comes family….

T: I would like to write it down on a piece of paper so that we can visualize it. So, this line here is a time line, and here, is where there is some changes in your life. So, I would like to focus about goals. So, this line here, can we say this is before you had children and this is after?

P: Okay.

T: So, what was your aspirations before you had children?

P: I wanted to be a obstetrician.

T: Ok, let us have it down on paper here….obstetrician. What aspire you to be one?

P: When I was younger, I watched a movie about the life of a doctor helping women with difficult deliveries in a village. Since then, I have always wanted to do the same.

T: So you were motivated to help women. You wanted to be a doctor because you were motivated by…?

P: By charity, helping others.

T: Charity. Can we have that written down on the time line?

P: Yes. If I do go back to doing clinical work with flexible hours, then I will have more time to do charity work.

T: Great! What kind of charity work do you have in mind?

P: I was thinking maybe volunteering at a free clinic. I did that once many years ago. I could not continue doing it then. If I have more flexible working hours, I am hoping that I will be able to do it again.

T: Sounds like a plan. Ok, let us have it written down. So, looking at this, it does not mean that you are not able to do what you dream about altogether. You could still do what you like, just in a slightly different setting, but you will be still helping people.

P: In terms of helping people…yes. But the career path is different.

T: Yes. So, if we take that and put it into the time line, you are not totally giving up on what aspire you.

P: Yeah. I guess you are right.

T: Is there any other way that you will be able to help others with your medical knowledge?

P: Yes, well…..I guess I can work in a local clinic as the hours are more flexible and I do not have to be on call. But I know I am ‘dragging' it and not setting a date to actually do it.

T: Do you know what is stopping you from doing it?

P: Yes….I have not been doing clinical work for a long time. I am not sure if I still have the skills.

T: I think it is normal to feel that way. I supposed it will be like the first day you were working as an intern many years ago. Let us make a mark on the time line here, first day as an intern. What was it like for you then?

P: Oh yes. I was feeling nervous.

T: So how did you managed your nerves?

P: Well, I have friends who did it with me.

T: So you have friends who gave you support. Let us write it down on the time line. How would you be able to adopt how you have coped then to help you now?

P: Well. I actually have a friend who had a similar experience as me. I suppose I can ask her about her experience.

T: That's good.

P: But do you think this is the right thing for me to do, going back into clinical work?

T: Well, to be honest, I do not have an answer for you. But we have had quite a number of sessions for me to get to know you better. What I gathered is that doing clinical work is something that you have enjoyed doing in the past, but you have to stop doing it for a while because of new priorities, your family and children and I just feel like if you do not try to go back to clinical work, I'm afraid that it will be something you would regret and kept you thinking, ‘What if I had tried, if I had gone and do it.' That's what my take of it. What do you think?

A: I think if that I do not try, I'll just be stuck in this viscous cycle and I'll be unhappy. So I want to stop this cycle.

B: Yeah, I think that's a good reason to do it. So maybe the homework for today is to adopt how you have cope previously, which is reaching out to friends for support, and to use that to help you in this current situation.

A: You're right.

#### 2.2.7. Session 7

She was pleased to report that she had undertaken some reading to refresh her knowledge and that had boosted her confidence. So she was able to approach the local clinic for a job interview and was offered employment. Her working hours were flexible, and she was given time to pick up her children from kindergarten. It was a dream come true for her. However, she found out that her husband had agreed to childcare arrangements suggested by her in-laws without prior discussion with her. This resulted in an argument with her husband that left her feeling angry and disappointed. Analysis of this interpersonal incident was done, and a more adaptive style of communication was role played during the therapy session. During role play, she learned that it was helpful to express her feelings using I-statement. (“I did not feel respected when you did not discuss childcare decisions with me before making decisions. I hope we can discuss this matter and come to a decision together.” rather than “You did not care about my feelings. How could you decide on that before even talking to me?”) She had decided to make arrangements to talk to her husband about childcare arrangements in the following week. She felt the best time to have a conversation with her husband was after dinner in the coming weekend.

#### 2.2.8. Session 8

She reported that the conversation with her husband about childcare arrangements went well. She was able to express her needs for him to discuss important matters with her to arrive to a collaborative decision. He had also agreed to inform her in-laws about their collective decision on childcare. This gave her a great sense of relief. In this session, she conveyed her needs to speak to her husband to help bath her children in the morning on weekends. She mentioned that he had started helping out in the past when she requested him to do so. However, as time goes by, the responsibility seemed to fall back to her. Role play on adaptive conversation on this matter was done in the session.

#### 2.2.9. Session 9

She had a conversation with her husband and requested him to help bath her children at the weekends. The conversation with her husband had allowed her to understand her husband's need to sleep in on weekends and thus explained his preference to bath the children later in the afternoon. They were able to negotiate a mutual agreement and the conflict was resolved. In addition, the patient realized that she had the tendency to jump to conclusion that her husband was not willing to help her without considering the needs of her husband. This is an important revelation that may help reduce future interpersonal conflict between the patient and her husband.

### 2.3. Concluding Sessions

She continued to report improvement in depressive symptoms over the next few sessions with no major IPT issues. The initial goals of IPT of was reviewed. She reported improvement of relationship with her husband and she felt more confident to start doing clinical work in a general practitioner clinic therefore concluding that the goals of IPT were achieved. Discussion on possible relapse of symptoms and plan for action when relapse was done. She was encouraged to share her feelings about concluding acute IPT sessions. She found IPT very helpful. She was happy to conclude acute sessions and continued maintenance sessions.

### 2.4. Outcome and Follow-Up

At the conclusion of her acute IPT treatment, she was able to manage her two older children and newborn with the help of her husband. She felt more empowered to start a career in clinical work. Her depression improved, and she was able to achieve remission of symptoms.

## 3. Discussion

Perinatal depression is a depressive disorder occurring in women during pregnancy or postnatal period [13]. The prevalence of perinatal depression varies across different countries. The prevalence of perinatal depression in higher-income countries ranges from 7 to 15%, while prevalence in low- and middle-income countries is noted to be twofold higher [14, 15]. Studies indicate that one in four women in South Asian countries suffered from depression during perinatal period [16].

Perinatal depression causes considerable social, economic, and health care burden on women, their partners, extended family, and children [17]. The impact is noted to be higher on women in low- and middle-income countries [15]. In addition to biological vulnerability to major depression, low social support, interpersonal conflict, and difficulties in role transition and gender inequality are important risk factors leading to perinatal depression [18–20].

IPT is an effective psychotherapy treatment for perinatal depression, because it targets specific interpersonal factors related to depression in women during the perinatal period [21]. Specifically, IPT focuses on mobilizing social support for the smooth transition into motherhood. As demonstrated in this case report, IPT was effective for this patient in dealing with her interpersonal conflict and pursuing her career.

The recommended structure for acute IPT treatment is at least 12 sessions of therapy. However, therapy should be tailored to fit the patient's needs rather than forcing the patient into a rigidly structured therapy [22]. Therapeutic collaboration that takes into account the patient's needs is an important factor that predicts therapeutic outcomes [23]. In this case, the patient showed great improvement in symptoms and return of normal functioning level following 9 sessions of acute treatment, and she was comfortable to move forward to concluding and maintenance sessions. Nevertheless, research study demonstrated acute treatment is not sufficient to prevent relapse [21]. Thus, maintenance IPT sessions following acute sessions are needed to prevent future relapse.

One of the focuses of IPT in this case is to deal with interpersonal dispute the patient had with her husband. In IPT, interpersonal dispute is resolved by helping the patient to express needs and wishes through modelling of adaptive communication in therapy session. A resolution is successfully achieved when an understanding is met with some compromise on both part. In the recurrent pattern of interpersonal dispute, IPT involves helping the patient to gain awareness of unspoken assumptions and nonreciprocal expectations related to the dispute. This was demonstrated in this case.

Since the mid-1970s, there has been a shift towards a more egalitarian view of the roles and responsibilities of a married couple [24]. This has led to an increased degree of responsibility assumed by working married women, and this correlated with higher incidents of interpersonal conflicts within couples. Women in dual-earning families experienced pressures from the demands of multiple roles and responsibilities associated with work and family. Many work and family conflicts arise from issues concerning gender ideology and household task division [25]. Gender ideology is defined as beliefs regarding normal roles and responsibilities of men and women in a marriage [25, 26]. Research findings show that husbands with traditional gender ideology are less likely to share the burden in childcare and household chores leaving their wives unsupported in their struggle to find balance between the demands of work and family roles [25, 26]. Gender inequality in household task division is found to be related to relationship dissatisfaction and relationship dissolution [27].

The difficulties faced by working women in handling work-life balance are clearly demonstrated in this case. The patient was forced to give up her career as a doctor after marriage as her husband and in-laws did not approve of her working night shifts. Her husband relied on her on childcare and many household chores, thus increasing the level of stress she faced. She was also worried about expressing her difficulties due to depression as a result of fear of overinvolvement of her in-laws. Relationships with in-laws play an important role in a marriage. The type and quality of the in-law relationship can be a source of stress or support for the couples and thus has an impact on marital satisfaction and quality of life [28]. Conflict may arise if there is a difference between in-laws and couples with regards to traditional versus egalitarian views of autonomy and boundaries in a marriage [29].

In conclusion, interpersonal dispute and difficulties in role transition are common factors for perinatal depression. IPT is empirically proven to be effective for treatment of peripartum depression.

## Data Availability

The data used to support the findings of this study are included within the article.

## Additional Points

*Learning Points*. Interpersonal disputes between couples and the role transition into motherhood and multiple roles of working women following delivery are important risk factors to consider for perinatal depression. Interpersonal psychotherapy (IPT) is an effective treatment for women suffering from perinatal depression as a result of these factors. IPT focuses on improving communication and adopting practical adaptive responses via role play during therapy session.
